# Predictors of Mortality in Preterm Infants with Respiratory Distress Syndrome: A Retrospective Analysis

**DOI:** 10.3390/jcm15020691

**Published:** 2026-01-15

**Authors:** Lovro Vrakela, Branka Polić, Dina Mrčela, Joško Markić, Tatjana Ćatipović Ardalić, Tanja Kovačević, Zenon Pogorelić

**Affiliations:** 1 School of Medicine, University of Split, Soltanska 2, 21000 Split, Croatia; 2 Department of Pediatrics, University Hospital of Split, Spinciceva 1, 21000 Split, Croatia; 3 Department of Pediatric Surgery, University Hospital of Split, 21000 Split, Croatia

**Keywords:** preterm infants, RDS, intensive care, surfactant, ventilation

## Abstract

**Aims:** The aim of this study was to evaluate clinical outcomes and identify predictors of mortality in preterm infants with respiratory distress syndrome (RDS) treated in a tertiary Pediatric Intensive Care Unit (PICU). **Methods**: This retrospective study included 86 preterm infants diagnosed with RDS and treated between January 2015 and December 2024. Clinical data were extracted from medical records and included demographic and anthropometric parameters, perinatal history, associated neonatal diagnoses, ventilation type and duration, surfactant administration, use of inotropes and antibiotics, cranial ultrasound findings, and PICU length of stay. **Results**: Mortality was 18.6%, with the highest rates observed in extremely preterm infants (<28 weeks) and those with extremely low birth weight (<1000 g). Several clinical variables were significantly associated with survival: gestational age, birth weight, birth length, and Apgar scores at 1 and 10 min (all *p* ≤ 0.005). In multivariable logistic regression, each additional week of gestation (OR 0.72, 95% CI 0.59–0.87), higher birth weight (OR 0.998, 95% CI 0.997–0.999), and higher Apgar scores (OR 0.69 at 1 min; OR 0.60 at 10 min) were significantly associated with survival. Ventilation was required in 97.7% of infants, and outcomes differed significantly by ventilation modality (*p* = 0.021), with the lowest mortality observed in those treated with combined invasive and non-invasive ventilation. Resuscitation (*p* < 0.001) and inotropic support (*p* < 0.001) were strongly associated with death. Length of PICU stay and duration of ventilation were significantly shorter in non-survivors (*p* < 0.05). Surfactant therapy was used in 79.1% of infants but was not significantly associated with survival. **Conclusions**: Gestational age, birth weight, and early postnatal condition were the strongest predictors of survival in preterm infants with RDS. Non-invasive and combined ventilation were associated with better outcomes, whereas the need for resuscitation and inotropes indicated markedly higher mortality. These results highlight the importance of early stabilization and optimized respiratory support in improving outcomes.

## 1. Introduction

Respiratory distress syndrome (RDS) is one of the most commonly diagnosed conditions in neonates treated in intensive care units. Historical names for RDS, such as hyaline membrane disease and pulmonary hyposurfactosis, reflect its pathophysiology and etiology, primarily caused by insufficient pulmonary surfactant production and lung immaturity [1]. RDS also increases the risk of developing other life-threatening conditions. In Denmark in 2016, Thygesen et al. showed that moderate and late preterm infants with RDS had an almost fourfold higher risk of cerebral palsy, in addition to increased rates of intraventricular hemorrhage and periventricular leukomalacia [2]. Mortality correlates with the level of development of the healthcare system: in the past, mortality in the United States was almost 60% and even higher in poorer countries, although modern therapeutic measures have significantly reduced it [3].

The incidence of RDS is estimated to be less than 3% in the total neonatal population, but is much higher in preterm infants compared with those born after 37 weeks of gestation. The incidence is highest among extremely preterm infants with gestational age less than 28 weeks (60–80%) [4]. This is largely because functional and sufficient surfactant levels in the alveoli are typically not present until around the 35th week of gestation [5]. According to the Croatian Institute of Public Health, 32,674 children were born in Croatia in 2023, and 0.35% of them were born before the 28th week of gestation [6].

The leading pathophysiological mechanism in RDS is the lack of pulmonary surfactant, a thin layer of phospholipids and lipoproteins that reduces alveolar surface tension and facilitates alveolar expansion. Surfactant production in type II pneumocytes begins around the 24th to 28th week of gestation, and by the 35th week, alveoli contain levels similar to mature lungs [5]. For this reason, gestational age is considered the key risk factor for RDS. Other major risk factors include low birth weight, male sex, white race, cesarean delivery, chorioamnionitis, fetal hydrops, gestational diabetes mellitus (GDM), and familial genetic predisposition [7]. Male infants have been shown to have a 1.87-fold higher risk of developing RDS [8]. Cesarean delivery is another well-established risk factor because hypersecretion of catecholamines and endogenous corticosteroids—important stimulators of surfactant production—is absent during cesarean birth [9]. Protective factors include intrauterine growth restriction (IUGR), prolonged rupture of membranes, and antenatal corticosteroid therapy. Administration of antenatal corticosteroids significantly reduces both the incidence and mortality of RDS [10].

The clinical presentation of RDS develops rapidly after birth. Symptoms include tachypnea, grunting, intercostal retractions, and cyanosis, with typical progression within the first 48 h of life [11]. Diagnosis requires exclusion of infection, identification of the characteristic radiological finding of reticulogranular lungs with reduced aeration, and recognition of increased oxygen requirements within the first 24 h [12]. Radiographically, a diffuse “ground-glass” appearance with reduced lung volume is typical. In the last decade, lung ultrasound has gained importance as a rapid, simple, non-invasive, and radiation-free diagnostic tool [13].

Rapid diagnosis and early management immediately after birth are crucial. In severe cases with failure of vital functions, resuscitation, oxygen administration, and continuous monitoring are necessary. If no improvement occurs, the newborn should be transferred to the ICU for intubation, ventilation, and surfactant therapy [14]. Corticosteroids are central to prenatal management, and even a single dose before 34 weeks of gestation has been shown to improve fetal survival [15]. Beyond reducing mortality, antenatal corticosteroids significantly lower the overall incidence of RDS and decrease the likelihood of severe forms of the disease [16].

One of the most important modalities of RDS treatment is the administration of exogenous surfactant, which improves survival and reduces pneumothorax risk, especially in preterm infants [17]. Two main methods exist: the traditional IN-SUR-E (Intubate-Surfactant-Extubate) technique, and the newer, less invasive LISA (Less Invasive Surfactant Administration), in which Continuous Positive Airway Pressure (CPAP) is maintained while surfactant is delivered through a thin catheter [18,19]. Numerous advantages of non-invasive ventilation over invasive ventilation—particularly the reduced risk of lung injury—have been demonstrated [20]. Consequently, CPAP has been the primary method for treating preterm infants with RDS for more than 50 years, and is used across both high-income and low-income settings [21].

In this study, we retrospectively analyzed clinical, perinatal, and therapeutic factors associated with outcomes in preterm neonates diagnosed with RDS treated at the Pediatric Intensive Care Unit of the University Hospital of Split. Despite well-established prognostic factors, long-term real-world data from tertiary intensive care settings remain limited, particularly regarding combined respiratory strategies. This study aimed to evaluate clinical outcomes and identify predictors of mortality in preterm infants with respiratory distress syndrome (RDS) treated in a tertiary Pediatric Intensive Care Unit, without prespecifying individual prognostic factors. While individual prognostic factors are well established, their combined impact in real-life tertiary intensive care settings, particularly in relation to dynamic ventilation strategies, remains insufficiently characterized.

## 2. Methods

### 2.1. Patients

The study was conducted at the Department of Pediatric Intensive Care Unit (PICU), University Hospital of Split, Croatia. Medical records of all preterm neonates diagnosed with RDS and hospitalized between 1 January 2015 and 31 December 2024 were reviewed. A total of 86 patients were included in the study. Inclusion criteria were: clinical manifestation and confirmed diagnosis of RDS, gestational age < 37 weeks, and admission to the PICU. In our institution, the Pediatric Intensive Care Unit is used for managing preterm neonates requiring advanced respiratory or hemodynamic support from other hospitals, while the Neonatal Intensive Care Unit is reserved only for preterm neonates born in the University Hospital of Split.

Exclusion criteria were patients in whom the diagnosis of RDS was not confirmed, term neonates, and patients with missing data in medical records. Patients with missing key clinical data were excluded from the respective analyses, and no data imputation was performed.

### 2.2. Ethical Considerations

The study protocol was reviewed and approved by the Ethics Committee of the University Hospital of Split (Approval number: 520-03/25-01/64; date of approval: 20 February 2025). The study was carried out in accordance with the principles of the Declaration of Helsinki and institutional data protection guidelines.

### 2.3. Study Design

Data were collected retrospectively from both electronic and paper medical records and included demographic, anthropometric, perinatal, clinical, and outcome variables. Demographic data comprised information on sex, place and time of birth, while anthropometric parameters included gestational age, birth weight, birth length, and Apgar score. These parameters were compared between the two analyzed groups—patients who had a positive outcome (*n* = 70) and patients who died (*n* = 16).

Perinatal history was reviewed to identify maternal and pregnancy-related conditions such as GDM, preeclampsia, intrauterine infection, premature rupture of membranes, IUGR, pathological cardiotocography (CTG), placental abruption, and uterine abnormalities, including placenta previa, bicornuate uterus, or uterine myoma. Pre-existing chronic maternal diseases diagnosed before pregnancy were also documented. In addition to RDS, the presence of other comorbidities or associated neonatal diagnoses was recorded, including cardiopulmonary failure, perinatal hypoxia, early-onset neonatal sepsis, Edwards syndrome, anal atresia, pneumothorax, perinatal asphyxia, pneumonia, perinatal infection, intracranial hemorrhage, hydrocephalus, anemia of prematurity, congenital cardiac anomalies such as patent ductus arteriosus or tetralogy of Fallot, and gastroschisis.

Therapeutic and clinical data included the need for resuscitation, administration of surfactant, type and duration of ventilation, antibiotic therapy, use of inotropic agents and inhaled nitric oxide (iNO), and the requirement for parenteral nutrition. Cranial ultrasound findings were reviewed, and intracranial hemorrhages were graded using the Papile classification. The duration of stay in the intensive care unit and survival status at discharge were recorded as outcome measures.

### 2.4. Outcomes of the Study

The primary outcome of this study was mortality during PICU hospitalization among preterm infants with RDS, analyzed in relation to demographic factors, perinatal characteristics, and key treatment interventions, such as resuscitation, respiratory and inotropic support, parenteral nutrition, and admission of antibiotics. Secondary outcomes included the type and duration of ventilation, need for resuscitation at birth and inotropic support, administration of surfactant therapy, patterns of antibiotic use, cranial ultrasound findings, length of PICU stay, and the association of comorbidities and complications with survival. Mortality was defined as death occurring during PICU hospitalization, without differentiation between early and late neonatal death. All outcomes were assessed using univariate and multivariate statistical analysis to identify independent predictors of mortality and treatment response in preterm infants with RDS.

### 2.5. Statistical Analysis

Statistical analysis was performed using Python software (version 3.11) within the JupyterLab environment and Statistical Package for the Social Sciences (IBM SPSS software, version 28). Categorical variables were presented as absolute frequencies and percentages, and differences between outcome groups were assessed using the Chi-square test or Fisher’s exact test, depending on expected cell counts. The normality of continuous variables was assessed using the Shapiro-Wilk test. All continuous variables showed a non-normal distribution, and were presented as medians with interquartile ranges (IQR) and compared using the Mann-Whitney U test. To explore the association between continuous variables and neonatal outcomes, binary logistic regression analysis was conducted. Variables were entered into the multivariate logistic regression model based on clinical relevance and statistical significance in univariate analyses. A two-tailed *p*-value < 0.05 was considered statistically significant. All graphical representations were generated using Python libraries such as matplotlib and seaborn, ensuring consistent visualization of data and statistical trends.

## 3. Results

In the selected study period, a total of 86 preterm infants with RDS were treated at the PICU, University Hospital of Split. The highest number of hospitalized preterm infants was recorded among those born in 2016 and 2022, with eleven cases in each year. The general demographic and anthropometric characteristics of all subjects, as well as stratification according to survival outcome, are presented in Table 1.

A statistically significant difference in survival was observed with respect to gestational age (*p* = 0.001), with markedly lower survival rates among infants with lower gestational ages. The poorest outcome was noted among extremely preterm infants (*n* = 16), whose survival rate was 50%. Survival increased progressively with advancing gestational age. Among those born between 31 and 34 weeks, the survival rate reached 84%, whereas all infants born between 35 and 37 weeks survived (100%). Throughout the study period, male infants predominated, accounting for 56 cases (65.2%), compared to 30 female infants (34.8%). No statistically significant difference in outcome according to sex was observed (*p* = 0.593). Birth weight was significantly associated with outcome (*p* < 0.001), with a higher number of deaths recorded among infants with lower birth weights. The highest mortality (58.3%) was observed among infants with birth weight <1000 g, followed by 25% mortality among those weighing 1000–1499 g. The best survival rate was found in infants weighing >2500 g (95.2%), while a high survival rate of 90.9% was also recorded among those weighing 1500–2499 g. Regarding other anthropometric measures, low survival was also noted among infants with body length <40 cm, where the mortality rate reached 58.6%. A statistically significant difference was confirmed across groups when comparing survival by body length (*p* < 0.001), with the highest mortality among infants shorter than 40 cm. An Apgar score of 7–10 at one minute was recorded in 45 infants (52.3%), and at ten minutes in 53 infants (61.6%). Statistically significant differences in both the 1- and 10-min Apgar scores were observed in relation to survival, indicating a higher mortality rate among infants with lower scores (1–7) (*p* = 0.005). Among non-survivors, an Apgar score of 1–7 was recorded at one minute in 10 infants (31.2%) and at ten minutes in 5 infants (41.7%).

Comparison of clinical characteristics and treatment modalities is shown in Table 2.

A statistically significant difference in outcome was observed among infants who underwent cardiopulmonary resuscitation (CPR), with a higher mortality in the resuscitated group compared to those not resuscitated (15 [71.4%] vs. 1 [1.5%], *p* < 0.001). Only two infants (2.3%) were not mechanically ventilated during hospitalization. Regarding the type of ventilatory support, the majority (39 infants, 45.3%) received both invasive and non-invasive ventilation during treatment. Exclusive invasive ventilation was used in 29 infants (33.7%), whereas 16 infants (18.6%) required only non-invasive ventilation. Survival rates differed significantly depending on the type of ventilation (*p* = 0.021). The highest survival was recorded among infants who received both invasive and non-invasive, while the lowest survival occurred in those ventilated exclusively invasively. A statistically significant difference was also observed in the duration of ventilation between survivors and died (*p* = 0.047).

The median duration of ventilation among survivors was six days, compared with 2.5 days among those who died. The length of stay in the PICU was significantly longer for survivors compared to non-survivors (21 vs. 3 days, *p* < 0.001). Surfactant therapy was administered in 68 infants (79.1%). No statistically significant difference in survival was found between infants who did and did not receive surfactant therapy (*p* = 0.505).

Regarding antibiotic therapy, 41 infants (47.7%) received first-line antibiotics (ampicillin and gentamycin), 11 (12.8%) received second-line or reserve antibiotics (vancomycin and meropenem), and 29 (33.7%) received a combination of both lines. These regimens were generally associated with survival rates above 80%. However, the use of reserve antibiotics (amikacin, teicoplanin, ceftriaxone, piperacillin-tazobactam, sulfamethoxazole-trimethoprim) and antifungal therapy (micafungin), typically prescribed in severe infections and sepsis, was linked with higher mortality, though without statistical significance (*p* = 0.157). Inotropes were used in 40 infants, of whom 16 (40%) died.

No significant difference in survival was observed regarding parenteral nutrition (*p* = 0.204) (Table 2). On cranial ultrasound, 52 infants (60.5%) showed increased parenchymal echogenicity, with an 88.5% survival rate. Periventricular cysts were found in 17 infants, with a survival rate of 94.1%. The lowest survival (66.7%) occurred in infants with cerebral malformations and those with normal ultrasound findings, though the number of such cases was small. Most infants (*n* = 47) had no evidence of intracranial hemorrhage (ICH), while grade II hemorrhage was the most common (23 infants, 26.7%) type of hemorrhage, and both groups showed high survival. Grades III and IV were associated with mortality rates of 40% and 33.3%, respectively (Table 2).

Table 3 summarizes the results of binary logistic regression for selected clinical variables (gestational age, birth weight, body length, Apgar scores at 1 and 10 min) in relation to mortality. Effect sizes are presented as odds ratios with 95% confidence intervals to facilitate clinical interpretation. Given the limited number of mortality events, the logistic regression results should be interpreted with caution and primarily as exploratory estimates of association.

Increasing gestational age significantly reduced the risk of death (β = −0.332, OR = 0.718; *p* = 0.001), as did higher birth weight (β = −0.002, OR = 0.998; *p* = 0.001) and body length (β = −0.23, OR = 0.792; *p* = 0.001). Higher Apgar scores at 1 and 10 min were independently associated with reduced mortality risk (Apgar 1 min: β = −0.373, OR = 0.689, 95% CI 0.565–0.855, *p* = 0.001; Apgar 10 min: β = −0.528, OR = 0.598, 95% CI 0.411–0.845, *p* = 0.004).

**Figure 1:**
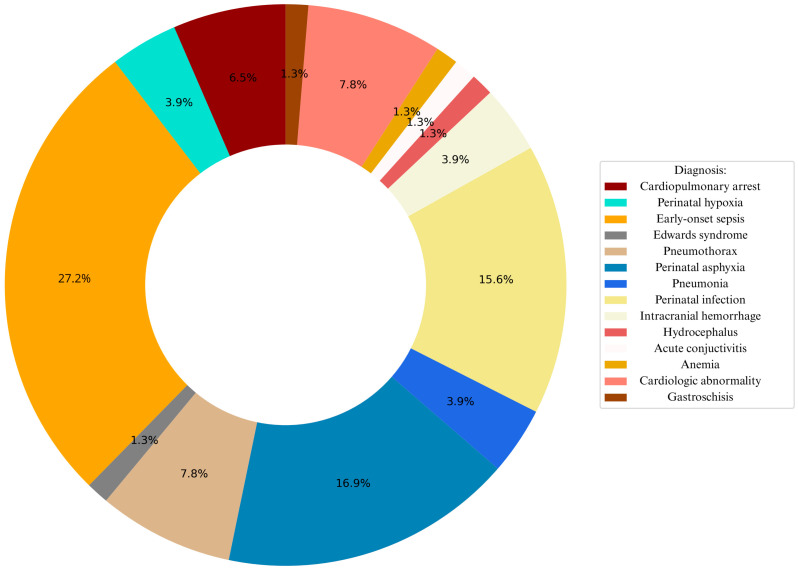
Graphical representation of leading diagnoses associated with RDS.

Figure 1 summarizes the most frequent diagnoses associated with RDS, including early-onset sepsis, perinatal asphyxia, pneumonia, perinatal infection, pneumothorax, congenital heart disease, cardiopulmonary failure and intracranial hemorrhage. Together, these conditions accounted for more than 74% of all comorbidities in the cohort, with cardiopulmonary failure demonstrating the highest mortality. Perinatal history revealed that 43% of pregnancies were uneventful, while complications such as preterm premature rupture of membranes (PPROM), gestational diabetes mellitus (GDM), and placental abruption were the most common among the remainder.

**Figure 2:**
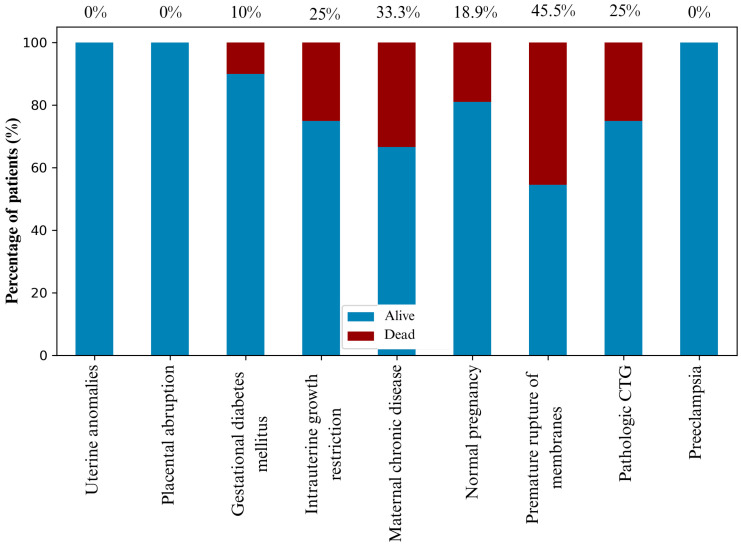
The probability of mortality outcomes based on the perinatal history of newborns, with exact values depicted above the columns.

Figure 2 illustrates mortality probability in relation to key perinatal factors. PPROM, maternal chronic disease and IUGR were associated with the highest mortality risk, while uncomplicated pregnancies showed the lowest risk pattern.

**Figure 3:**
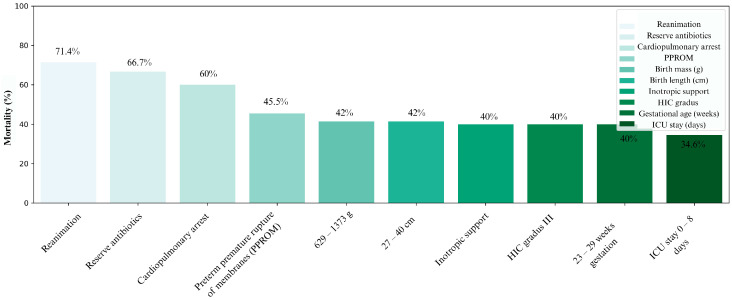
Distribution of all conditions according to the highest mortality, with exact values depicted above the columns.

Figure 3 illustrates ten key factors with the strongest negative impact on survival, considering all collected variables (excluding those with fewer than three cases). These include therapeutic modalities typically applied in critically ill neonates—resuscitation, reserve antibiotics, and inotropic therapy—as well as diagnoses such as cardiopulmonary failure, grade III ICH, and perinatal PPROM. Among anthropometric variables, the highest mortality was observed in infants with birth weight 629–1373 g, body length 27–40 cm, and gestational age 23–29 weeks. A shorter PICU stay was also associated with higher mortality.

## 4. Discussion

The obtained results confirmed that the incidence of RDS is highest among extremely preterm infants (<28 weeks) and those with extremely low birth weight (<1000 g), which is consistent with previous findings and data from the literature [22,23]. The analysis by gestational age and birth weight showed that these parameters are the most important predictors of poor outcome and mortality in preterm infants. Infants born before 28 weeks and/or weighing less than 1000 g had a significantly higher incidence of RDS, a greater need for ventilation, and higher mortality rates, thereby confirming the findings of the World Health Organization and large multicenter studies [24]. In our population, mortality among infants born <28 weeks of gestation was 50%, while in the group of 28–30 weeks it was 18.2%, which corresponds to trends observed in other European centers, where mortality among infants born before 28 weeks ranges between 25–40%, and for those between 28–31 weeks between 8–15% [25].

Our results further confirmed that higher gestational age, birth weight, birth length, and higher Apgar scores at 1 and 10 min were significantly correlated with higher survival rates, thereby confirming the hypothesis on the positive association of these factors with treatment outcome. Apgar scores at 1 and 10 min remained independent predictors of mortality in multivariate analysis, underscoring the importance of early postnatal condition beyond gestational age and birth weight. Similar findings were reported by Zeitlin et al., who noted that survival increases with higher gestational age, reaching more than 80% in infants older than 28 weeks [25]. The high mortality (41.7%) observed among infants with Apgar scores <7 at 10 min can be explained by the failure of initial therapeutic modalities or by deterioration within the first ten minutes of life [26]. Although the majority of participants (79.1%) received surfactant therapy, the analysis did not show a statistically significant association between its administration and survival. However, this finding should be interpreted cautiously, as data on timing of administration, dosage, number of doses, and method of surfactant delivery were not available and may represent important confounders. This finding also likely reflects confounding by indication, as surfactant is preferentially administered to more severely ill infants, which may mask its true therapeutic benefit.

However, non-invasive and combined (invasive + non-invasive) ventilation were associated with higher survival rates (92.3%) compared to exclusively invasive ventilation, which had a lower survival rate (65.5%). These findings indicate that the type and quality of ventilatory support play a greater role in survival than surfactant administration alone. Even though our data did not demonstrate a significant correlation between surfactant therapy and outcome, numerous studies have confirmed its effectiveness—Halliday et al. showed as early as 1990 that prophylactic surfactant administration significantly reduces mortality due to RDS and bronchopulmonary dysplasia, while Bevilacqua et al. demonstrated better outcomes in the prophylactic group compared to the selective one [27,28]. The Cochrane review from 2012 further confirmed that early surfactant administration, within two hours of birth, in combination with CPAP, significantly reduces mortality and the incidence of pneumothorax [29]. These associations should be interpreted cautiously, as confounding by indication cannot be fully excluded in this retrospective analysis. More intensive respiratory and pharmacological interventions were more frequently applied in the most severely ill infants, which may partially explain the observed associations with mortality.

The results concerning the type of ventilatory support were fully consistent with previous research. According to the Cochrane meta-analysis, the use of primary non-invasive ventilation (CPAP) reduces mortality, the incidence of BPD, and the need for intubation and later surfactant administration [30]. Similar advantages of non-invasive ventilation were confirmed by Ramaswamy et al., as well as by two Chinese prospective studies from 2020 and 2025, which showed that non-invasive high-frequency oscillatory ventilation (NHFOV) significantly reduces the need for transition to invasive ventilation [31,32,33]. In our cohort, the application of resuscitation procedures and inotropic support was significantly associated with a higher risk of mortality, supporting the hypothesis that more intensive therapy often reflects a more severe clinical condition [34]. The lower mortality observed in infants receiving combined invasive and non-invasive ventilation may reflect survivor bias rather than a distinct treatment strategy. Infants who survived long enough to transition from invasive to non-invasive support were more likely to be classified in this group.

The unexpectedly high mortality observed in infants with grade I intracranial hemorrhage likely reflects the presence of severe concomitant systemic illness, or occurred due to a very low sample size, rather than the hemorrhage itself.

Annamalai et al. demonstrated that infections caused by resistant bacteria increase mortality, whereas in our study, the type of antibiotics used was not significantly associated with outcome, likely due to the small sample size [35]. The length of PICU stay proved to be an important prognostic factor—infants who died had a median stay of three days, while survivors stayed an average of 21 days, suggesting that fatal outcomes most often occur in the early neonatal period. Although early versus late neonatal mortality was not analyzed separately, the markedly shorter PICU stay among non-survivors strongly suggests that most deaths occurred in the early neonatal period. These results are consistent with the findings of Lee et al. and Stoll et al., who reported that most deaths in infants <28 weeks occur within the first seven days, while infants with longer NICU stays have significantly lower mortality [36,37]. Shorter ventilation duration among non-survivors likely reflects early mortality rather than clinical improvement, as fatal outcomes often occurred before prolonged respiratory support could be established.

The main limitations of the study include the small number of participants in certain categories, its retrospective, single-center design, which limits generalizability and introduces a potential risk of selection bias, as well as residual confounding due to the inability to adjust for all clinically relevant variables. The lack of follow-up data on long-term neurological and respiratory outcomes further restricts the comprehensive assessment of the later development of surviving infants. In addition, clinically relevant comorbidities such as sepsis, pneumothorax, and congenital heart disease were not included in the multivariate analysis due to small subgroup sizes, which may have influenced the identification of independent predictors. The limited number of mortality events increases the risk of overfitting in multivariate analysis. Nevertheless, this study provides valuable insight into ten years of experience in the treatment of RDS at the University Hospital of Split, identifies key factors associated with survival, and highlights the importance of timely and individualized respiratory support in managing this high-risk population of preterm infants. Future research may also incorporate advanced digital and AI-based systems to improve risk assessment and early detection of complications in preterm populations. Recent evidence shows that even general-purpose large language models can provide clinically meaningful risk predictions in neonatal settings, such as identifying serious bacterial infections in young infants, highlighting their potential integration into neonatal intensive care practice [38].

## 5. Conclusions

This study highlights gestational age and birth weight as the strongest predictors of survival in preterm infants with respiratory distress syndrome (RDS). Extremely preterm and extremely low birth weight infants had the highest mortality, consistent with international data. Non-invasive and combined ventilation methods were associated with significantly better outcomes compared to exclusively invasive ventilation. Although surfactant therapy did not show a statistically significant effect, the quality of respiratory support proved crucial for survival. These findings emphasize the importance of early and individualized management of RDS and underline the need for future multi-center prospective studies to further refine treatment strategies and improve outcomes in this vulnerable population.

## Figures and Tables

**Table 1:** Demographic and anthropometric characteristics of preterm infants according to outcome.

	Total, n (%) (*n* = 86)	Survived, n (%) (*n* = 70)	Died, n (%) (*n* = 16)	*p*
Gestational age (weeks)				
<28	16 (18.6)	8 (50.0)	8 (50.0)	
28–30	22 (25.6)	18 (81.8)	4 (18.2)	0.001 *
31–34	25 (29.1)	21 (84.0)	4 (16.0)	
35–37	23 (26.7)	23 (100.0)	0	
Sex				
Male	56 (65.2)	47 (83.9)	9 (16.1)	0.593 *
Female	30 (34.8)	23 (76.7)	7 (23.3)	
Birth weight (g)				
<1000	12 (14.0)	5 (41.7)	7 (58.3)	
1000–1499	20 (23.3)	15 (75.0)	5 (25)	<0.001 *
1500–2499	33 (38.4)	30 (90.9)	3 (9.1)	
≥2500	21 (24.4)	20 (95.2)	1 (4.8)	
Birth length (cm)				
<40	26 (30.2)	14 (53.8)	12 (46.2)	<0.001 *
40–44.9	25 (29.1)	22 (88.0)	3 (12.0)	
≥45	32 (37.2)	31 (96.9)	1 (3.1)	
Apgar score (in 1st min)				
<7	32 (37.2)	22 (68.8)	10 (31.2)	0.005 ^†^
7–10	45 (52.3)	42 (93.3)	3 (6.7)	
Apgar score (10th min)				
<7	12 (14.0)	7 (58.3)	5 (41.7)	0.005 ^†^
7–10	53 (61.6)	48 (90.6)	5 (9.4)	

* Chi-square test, ^†^ Fisher’s exact test.

**Table 2:** Clinical characteristics and therapeutic modalities in hospitalized preterm infants.

	Total, n (%)	Survived, n (%)	Died, n (%)	*p*
Resuscitation, n (%)				
Yes	21 (24.4)	6 (28.6)	15 (71.4)	<0.001 ^†^
No	65 (75.6)	64 (98.5)	1 (1.5)	
Ventilation, n (%)				
Yes	84 (97.7)	68 (81.0)	16 (19.0)	>0.999 ^†^
No	2 (2.3)	2 (100.0)	0	
Type of ventilation, n (%)				
Invasive	29 (33.7)	19 (65.5)	10 (34.5)	
Non-invasive	16 (18.6)	13 (81.2)	3 (18.8)	0.021 *
Invasive+ non-invasive	39 (45.3)	36 (92.3)	3 (7.7)	
iNO, n (%)				
Yes	4 (4.7)	2 (50.0)	2 (50.0)	0.156 ^†^
No	82 (95.3)	68 (82.9)	14 (17.1)	
Surfactant, n (%)				
Yes	68 (79.1)	54 (79.4)	14 (20.6)	
No	18 (20.9)	16 (88.9)	2 (11.1)	0.505 ^†^
Antibiotics, n (%)				
Ampicillin + Gentamycin	41 (47.7)	34 (82.9)	7 (17.1)	
Vancomycin + Meropenem	11 (12.8)	9 (81.8)	2 (18.2)	0.157 *
First + second line therapy	29 (33.7)	25 (86.2)	4 (13.8)	
Other reserved antibiotics	3 (3.5)	1 (33.3)	2 (66.7)	
Inotropes, n (%)				
Yes	40 (46.5)	24 (60.0)	16 (40.0)	<0.001 ^†^
No	46 (53.5)	46 (100.0)	0	
Parenteral nutrition, n (%)				
Yes	65 (75.6)	55 (84.6)	10 (15.4)	0.204 ^†^
No	21 (24.4)	15 (71.4)	6 (28.6)	
Brain ultrasound, n (%)				
Hyperechoic	52 (60.5)	46 (88.5)	6 (11.5)	
Hyperechoic + cysts	17 (19.8)	16 (94.1)	1 (5.9)	
Brain malformation	3 (3.5)	2 (66.7)	1 (33.3)	
Normal	3 (3.5)	2 (66.7)	1 (33.3)	0.001 ^†^
Hemorrhage, grade I	3 (3.5)	0 (0.0)	3 (100)	
Hemorrhage, grade II	23 (26.7)	22 (95.7)	1 (4.3)	
Hemorrhage, grade III	10 (11.6)	6 (60.0)	4 (40.0)	
Hemorrhage, grade IV	3 (3.5)	2 (66.7)	1 (33.3)	
Length of ventilation, median (IQR)	6.0 (3.0–16.0)	6.0 (4.0–16.0)	2.5 (1.0–14.2)	0.047 ^‡^
Stay in intensive care unit, median (IQR)	14.0 (7.0–41.0)	21.0 (8.0–48.0)	3.0 (1.8–14.2)	<0.001 ^‡^

* Chi-square test, ^†^ Fisher’s exact test, ^‡^ Mann-Whitney U test; iNO—Inhaled nitric oxide, IQR—Interquartile range.

**Table 3:** Results of binary logistic regression analysis for selected clinical variables associated with mortality.

	Coefficient (β)	Odds Ratio (OR)	95% CI	*p* ^§^
Gestational age	−0.332	0.718	0.594–0.867	0.001
Birth weight	−0.002	0.998	0.997–0.999	0.001
Birth length	−0.233	0.792	0.700–0.896	0.001
Apgar 1st min	−0.373	0.689	0.565–0.855	0.001
Apgar 10th min	−0.528	0.598	0.411–0.845	0.004

^§^ Binary logistic regression.

## Data Availability

The data presented in this study are available on request from the corresponding author.
